# Characterization of a *WAS* splice-site variant in a patient with Wiskott-Aldrich syndrome

**DOI:** 10.3389/fimmu.2025.1517347

**Published:** 2025-01-23

**Authors:** Elisabetta Toriello, Rosa Maritato, Antonio De Rosa, Maria Valeria Esposito, Carla Damiano, Carmen Rosano, Emilia Cirillo, Antonietta Tarallo, Cosimo Abagnale, Francesca Cillo, Roberta Romano, Laura Grilli, Marika Comegna, Giancarlo Blasio, Giancarlo Parenti, Enrico Maria Surace, Giuseppe Castaldo, Claudio Pignata, Giuliana Giardino

**Affiliations:** ^1^ Department of Translational Medical Sciences, Pediatric Section, Federico II University of Naples, Naples, Italy; ^2^ CEINGE-Biotecnologie Avanzate Franco Salvatore S.c.a.r.l., Naples, Italy; ^3^ Telethon Institute of Genetics and Medicine (TIGEM), Pozzuoli, Italy; ^4^ Department of Molecular Medicine and Medical Biotechnologies, Federico II University of Naples, Naples, Italy

**Keywords:** Wiskott-Aldrich, WASP, splice-site mutation, next-generation sequencing (NGS), gene panel

## Abstract

Wiskott-Aldrich syndrome (WAS) (MIM #301000) is a rare X-linked primary immunodeficiency due to mutations in the *WAS* gene, characterized by thrombocytopenia with small platelets, eczema, recurrent infections, and an increased incidence of autoimmunity and malignancies. A wide spectrum of mutations has been identified in the *WAS* gene responsible for a broad variety of clinical phenotypes. By using targeted next-generation sequencing (t-NGS), we identified in a 2-month-old boy with thrombocytopenia and immunological alterations a 4-nucleotide deletion from position +3 to +6 of intron 8 (c.777 + 3_777 + 6delGAGT) of *WAS*, currently classified on ClinVar as a variant of uncertain significance. The *in-vitro* characterization of the variant revealed the complete retention of intron 8 in the mature transcript, suggesting a splicing defect due to the loss of a splice donor site at the 5′-end of intron 8. By sequencing the polymerase chain reaction product, we identified a premature stop at codon 269; thus, consequently, no Wiskott-Aldrich syndrome protein (WASp) was detectable in peripheral blood mononuclear cells from the patient. Due to the total absence of a full-length WASp, it is expected that the patient will develop the severe form of the disease, although further monitoring is needed to better define his phenotype.

## Introduction

Wiskott-Aldrich syndrome (WAS) (MIM #301000) is a rare X-linked primary immunodeficiency characterized by frequent infections, eczema, easy bruising and bleeding, thrombocytopenia, and small platelet size (microplatelets). Affected patients may also develop autoimmune disorders, such as hemolytic anemia or vasculitis, and they display an increased risk of specific types of cancer, particularly lymphomas (1, 2). The disease is caused by mutations in the *WAS* gene, located on the short arm of the X chromosome at position p11.22–p11.23. This gene is exclusively expressed in hematopoietic cell lineages, including T cells, B cells, natural killer (NK) cells, dendritic cells, macrophages, and platelets (3). WASp is a cytosolic protein comprising 502 amino acids that acts as a scaffold protein and transduces a wide range of signals from cell surface receptors to mediate dynamic changes in the actin cytoskeleton in response to external stimuli (4). In resting T cells, WASp is constitutively associated with WASp-interacting protein (WIP). WIP regulates WASp activity, promotes WASp stability by protecting WASp from degradation by calpain and proteasome, and it is also critical for localizing WASp to areas of actin polymerization (5–7).

To date, a wide spectrum of mutations has been identified in *WAS*, causing a broad variety of clinical phenotypes, ranging from isolated thrombocytopenia to classic WAS (8). *WAS* mutations include missense and nonsense, insertions, deletions, splice sites, and other complex mutations. Among these, missense mutations are typically located in the first four exons of the WASP homology 1 (WH1) domain and are the most common in patients with classic WAS. Conversely, splice site mutations are usually described within the C-terminal half of the gene, in introns 6–10, while deletions and insertions are reported through the whole gene (9). Several studies demonstrated that the severity of the clinical phenotype ranging from X-linked thrombocytopenia (XLT) to WAS is strongly influenced by the effect of the mutation on protein expression. In fact, the absence of WASp is typically associated with the severe WAS phenotype, while missense mutations or splice site mutations that result in reduced but still detectable WASp levels are usually found in patients with XLT (8, 10, 11).

In the present study, we investigate the pathogenic role of a splice site variant of uncertain significance (VUS) in the *WAS* gene, identified in a 2-month-old boy with thrombocytopenia and immunological alterations.

## Methods

### Mutation analysis

*T*-NGS analysis was performed using a panel of 141 genes associated with the main immunodeficiencies (list available upon request). For each gene, we analyzed the coding regions, 50 bp in each of the intronic boundaries, the promoter, and the 3′UTR for a total target size of about 1 Mb. The custom design of our probes was realized using the web-based SureDesign application (https://earray.chem.agilent.com/suredesign). A total of 50 ng of gDNA was processed through the SureSelectQXT Target Enrichment system (Agilent Technologies, Santa Clara, CA, USA) for Illumina-multiplexed sequencing. Sequencing reactions were carried out on the MiSeq instrument (Illumina, San Diego, CA, USA) using a PE 150 2 flow cell, running 16 samples for each sequencing run to obtain an average coverage of about 200 [>95% of the gene’s target nucleotides are covered at >100 reads, with mapping quality score (MQ >30) reads]; 96% of the analyzable target regions were covered by at least 50×. The Alissa Align & Call v1.0.2.10 tool (Agilent Technologies, Santa Clara, CA, USA), using the genome build hg38 as a reference, was used to perform alignments, variant calling, and quality filtering. The median QV bases used in variant calling were 39, with an average read length of 141 bp. Variant filtering and interpretation were done using Alissa Interpret v5.2.6 CE IVD software (Agilent Technologies, Santa Clara, CA, USA), using GRCh38.p2 and annotation sources like 1000 Genomes (Phase 3 release v5, 10 September 2014, including GRCh38 data), ClinVar (NCBI ClinVar October 2019), DGV (Database of Genomic Variants, version 15 May 2016), ESP6500 (variants in the ESP6500SI-V2 dataset of the exome sequencing project, annotated with SeattleSeqAnnotation137), ExAC (ExAC release 1.0—including GRCh38 from lif over data), OMIM (OMIM, version 25 October 2019), dbNSFP (dbNSFP v3.0b2: Database on functional predictions for nonsynonymous SNPs), dbSNP (dbSNP build 151), and gnomAD (gnomADrelease 2.0.2) (12).

### Blood cells

Whole blood from the affected patient, his mother, and unaffected control individuals was collected either in K3 potassium EDTA (1.8 mg/ml whole blood) or in lithium heparin (17 I.U./ml whole blood) anticoagulant agents and fractioned immediately. In order to obtain total blood white cells, samples collected in K3 potassium EDTA were treated twice with lysis buffer (10 mM Tris-HCl, 320 M sucrose, 5 mM MgCl2, 1& v/v Triton X-100, pH 8.0) to remove red blood cells and then centrifuged at 3000 rpm for 10 min. The pelleted white cells were then used for total RNA extraction. Peripheral blood mononuclear cells (PBMCs) were isolated from heparinized blood by HiSepTM LSM 1077 (HiMedia, Modautal, Germany) density gradient centrifugation with a standard protocol (13) and used for protein extraction.

### RNA extraction, reverse transcription, and PCR analysis

Total RNA was isolated from white blood cells using Trizol (Thermo Fisher Scientific, Waltham, MA, USA; Life Technologies) according to the manufacturers’ protocol. About 1 µg of total RNA was retrotranscribed with the high-capacity cDNA reverse transcription kit (Thermo Fisher Scientific, Waltham, MA, USA; Applied Biosystems) according to the manufacturers’ instructions. The obtained cDNA was used as a template in a polymerase chain reaction (PCR) with WASP-specific primers Fw: GCATGTCAGCCACGTGGGGTG and Rev: CCTGGCGCCTCATCTCCTGC. Standard PCR reactions (50 µl) were carried out with 0.2 mM dNTPs, 0.2 µm of each forward and reverse primer, 1.5 mM MgCl2, 1X PCR Buffer II (100 mM Tris-HCl, pH 8.3, 500 mM KCl) and 2.5 U AmpliTaq Gold DNA Polymerase (Thermo Fisher Scientific, Waltham, MA, USA; Applied Biosystems). PCR conditions were as follows: initial denaturation at 95°C for 5 min, 95°C for 30 s, 58°C for 30 s, and 72°C for 30 s for 35 cycles, and a final extension for 5 min at 72°C. The obtained PCR products were analyzed on a 2% agarose gel and revealed by UV irradiation.

### TOPO-TA cloning and sequence analysis

Following gel electrophoresis, the obtained bands from the patient and his mother were excised and the DNA was extracted from gel with the QIAquick PCR & Gel Cleanup Kit (Qiagen, Hilden, Germany) and cloned into the PCR 2.1 TOPO vector (Thermo Fisher Scientific, Waltham, MA, USA; Invitrogen). Positive white colonies were screened by minipreps (Qiagen, Hilden, Germany), digested with EcoRI endonuclease (New England Biolabs, Ipswich, MA, USA), and sequenced at the Eurofins Genomics facility with the primer Fw: CACAGGAAACAGCTATGACCATG, annealing on the vector, immediately upstream of the PCR insertion site.

### Protein extraction and Western blot analysis

PBMCs were lysed in RIPA buffer (50 mm Tris–HCl pH 8.0, 150 mm NaCl, 1% NP40, 0.5% Na- deoxycholate, 1 mm EDTA pH 8.0, 0.1% SDS). Lysis buffers were supplemented with protease inhibitors (Complete Protease inhibitor cocktail tablets; Roche, Milan, Italy) and 1 Mm phenylmethylsulfonyl. After lysis, total protein concentration was determined with the PierceTM BCA Protein Assay Kit (Thermo Fisher Scientific, Waltham, MA). Samples corresponding to 30 µg of protein lysate were denatured at 99°C for 5 min in 1× Laemmli sample buffer (Bio-Rad Laboratories, Hercules, CA, USA), then separated by SDS–polyacrylamide gel electrophoresis with a gradient 4%–15% polyacrylamide gel (#4568085 Bio-Rad Laboratories, Hercules, CA, USA) and transferred on a nitrocellulose membrane (#1704158 Transblot turbo, Bio-Rad Laboratories, Hercules, CA, USA). WASp and GAPDH were detected with mouse IgG2a anti-WASp mAb 5A5 (#557773 BD Biosciences, Allschwil, Switzerland) and IgG1 anti-GAPDH (#MA AM4300 Thermo Fisher Scientific, Waltham), respectively. Antibodies were diluted 1:1000 and 1:2000, respectively, in 5% milk in Tris-buffered saline with Tween 20 (0.05%), followed by incubation with 1:1000 secondary HRP-coupled antibody (Bio-Rad Laboratories, Hercules, CA, USA). Immunoreactive proteins were detected by chemiluminescence with ECL (Bio-Rad Laboratories, Hercules, CA, USA), and images were captured by ChemiDocTM XRS+ with ImageLabTM software. ImageLabTM software (Bio-Rad Laboratories, Hercules, CA, USA) was used for band densitometric quantification. For quantification, WASp bands were normalized to the endogenous GAPDH.

### q-real-time PCR

The PCRs with cDNA were carried out in a total volume of 20 μl, using 10 μl, LightCycler 480 SYBR Green I Master Mix (Roche, Switzerland), and 400 nM primers under the following conditions: pre-incubation, at 50°C for 5 min, cycling: 45 cycles of 95°C for 10 s, 60°C for 20 s, and 72°C for 20 s. Each sample was analyzed in duplicate in three independent experiments. The first q-real-time PCR was performed with primers pairs: Int8_FW: AGACCACTGCTGAGACCCCACC and Int8_RV: GGCGAGGAGACAAGCGACATGG; Ex5_FW: AGCTACCCCCACCACCAA and Ex7_RV: TATCAGCTGGGCTAGGTCCA. The second qRealTime PCR (G) was performed with primer pairs: Ex8_FW: TCAAGCATGTCAGCCACGTGGG and Ex9_RV: GTGAGCTGGGCCTCGCTGATTC; Int8_FW and Int8_RV.

## Results

**Figure 1:**
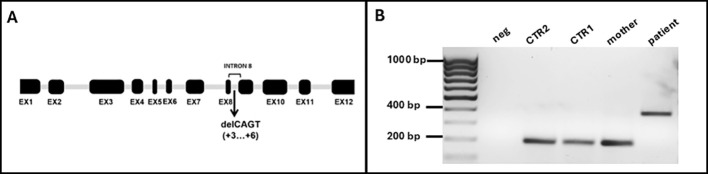
**(A)** Schematic representation of *WASP* gene with the new identified mutation in intron 8 (EX: exon; del: deletion). **(B)** Gel electrophoresis of the PCR products obtained from cDNA extracted from white blood cells using primers annealing on exon 8 (Fw) and exon 9 (Rev). neg: negative control; CTR2: male, young; CTR1: female, adult.

A 2-month-old boy presenting with petechiae and thrombocytopenia was admitted to our pediatric immunology center. He was born to nonconsanguineous parents. Familial history was negative except for thrombocytopenia not requiring treatment in the mother. Laboratory examinations, detailed in **Supplementary Table S1**, showed slightly increased eosinophil count and IgE levels, reduced CD8+ cells and IgM levels. The combination of clinical and laboratory features was suggestive of WAS. Flow cytometry analysis was performed to confirm the clinical suspect while waiting for the results of the genetic testing. It showed a significant reduction in WAS levels across four leukocyte populations compared to healthy control (CD16 + 22%, CD14 + 26%, CD3 + 12%, CD19 + 14%). *T*-NGS analysis followed by Sanger automated sequencing revealed a 4-nucleotide (nt) deletion from position +3 to +6 of intron 8 (c.777 + 3_777 + 6delGAGT) of *WAS* (NCBI ref. Number NM_000377.2) (**Figure 1A**), reported on the ClinVar database as a VUS. A subsequent trio NGS, performed at another institution, identified the same variant in the mother, whose history of thrombocytopenia had not been previously investigated.

Since this 4-nt deletion is located in intron 8, close to the exon (EX)-intron (INT) junction, we hypothesized that it could impair the normal splicing process due to the loss of a splice donor site, leading to the total or partial retention of INT8. To assess this hypothesis, we performed a standard PCR to amplify the region between EX8 and EX9 of the *WAS* transcript. We used *WAS-*specific primers and the cDNA obtained from total RNA extracted from a K3 Potassium EDTA-treated blood sample of the proband, his mother, and two healthy controls as a template (CTR1: adult, female; CTR2: young, male). A region of 226 bp from the housekeeping *GAPDH* was amplified from all samples as quality control of the samples (data not shown). Upon electrophoresis on a 2% agarose gel of the obtained PCR products, we observed a 400 base pair (bp)–long band in the proband, consistent with the retention of the 199 bp–long mutant INT8, while in his mother and in both healthy controls, we found a band of the expected size (197 bp) (**Figure 1B**). Notably, although the mother was known to be a carrier of the mutation, we could not detect the aberrant transcript in her sample. This is consistent with the finding that mature blood cells from female carriers of a defective *WAS* gene may show a nonrandom pattern of X-chromosome inactivation, with the chromosome bearing the normal *WAS* gene active in most cells (14, 15). A predictive analysis of the effect of the 4-nucleotide deletion on splicing, using the splice site prediction tool Spliceator, confirmed the loss of a putative splicing donor site immediately upstream of the 4-nucleotide deletion. Notably, by lowering the stringency of the analysis parameters (reliability = 50%), the activation of a putative splicing donor site within exon 8 is predicted with a score of 0.973. However, this would result in the exclusion of the entire exon 8 from the mature transcript, something that seems unlikely since the primer EX8 Fw used for cDNA amplification includes this sequence, and the primer actually pairs with the template to yield the amplification product.

**Figure 2:**
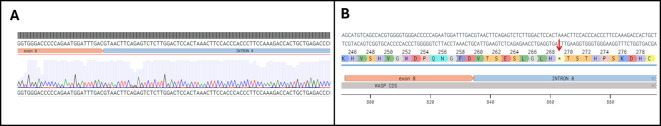
**(A)** Results of Sanger sequencing analysis performed on the PCR product from the affected patient showing the retention of intron 8 into the mature transcript. **(B)** Prediction of the effect of retention of intron 8 on protein traslation; *indicates the putative premature stop codon at position 269.

**Figure 3:**
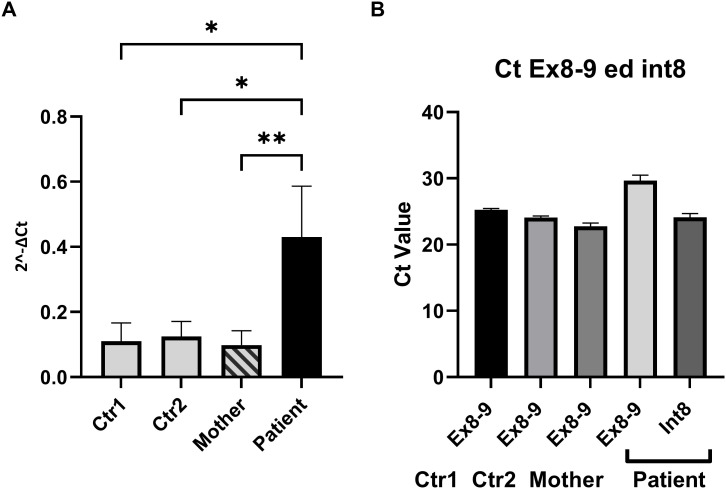
Quantitative analysis of transcript levels using RT-qPCR. **(A)** RT-qPCR was performed to compare the expression levels of the transcript containing retained intron 8 (INT8) in the proband (dark bar) to wild-type controls (light bars) and the carrier mother (striped bar). Primer pairs INT8Fw and INT8Rv were used to specifically amplify the transcript with retained intron 8, while the expression levels were normalized to the internal region of the WAS gene spanning exons 5–7 (Ex5–Ex7) for consistency across all samples. The results showed significantly higher expression of the INT8 transcript in the proband compared to controls and the carrier mother. The amplification observed in healthy controls, where intron 8 is normally spliced out, likely represents either background nonspecific amplification or low-level splicing artifacts. Error bars represent the mean ± standard deviation of three independent experiments. Statistical significance was assessed using ordinary one-way ANOVA (*p < 0.05; **p < 0.01). **(B)** Comparison of Ct values obtained using primer pairs INT8Fw/INT8Rv and Ex8Fw/Ex9Rv for the proband and Ex8Fw/Ex9Rv for controls and the mother. These primer sets amplify different regions of the WAS gene, specifically targeting the INT8 transcript or the canonical transcript. The Ct values were similar in magnitude across the samples, suggesting comparable levels of transcript abundance. However, the Ct values were not normalized against a common housekeeping gene, and the comparison assumes equal amplification efficiencies for the primer pairs. These efficiencies were validated through standard curve analyses, confirming their equivalence.

A direct Sanger sequencing analysis of the PCR product further confirmed that the longer band observed in the affected patient was produced as a result of the full retention of INT8 into the mature transcript (**Figure 2A**). Since splice site variants can sometimes result in the generation of alternative splice sites, the analysis was performed on the cDNA obtained from ten colonies from both the proband and his mother by restriction digestion followed by sequencing. Identical sequencing results were observed for all the screened clones. The insertion of INT8 into the mature transcript (**Figure 2B**) generates an aberrant open reading frame containing nine extra codons followed by a stop codon (at codon 269), leading to the production of a putative aberrant 268 amino acids–long protein. We then performed RT-qPCR on the extracted cDNAs to quantify in the proband the expression levels of the transcript containing the INT8. To normalize the data, we used an internal control as a reference gene (region spanning from EX5 to EX7 of the same gene for each sample), independent of the splicing site, rather than a housekeeping gene (16). As shown in **Figure 3A**, the transcript containing INT8 was expressed at higher levels in the proband compared to controls. This finding may support that nonsense-mediated mRNA decay (NMD) (17) is not operating to prevent the synthesis of the truncated protein. It should be noted that in the healthy controls, where intron 8 is normally spliced out, the INT8-containing transcript is not expected to be present. Thus, the amplification observed in controls (**Figure 3A**) likely represents either background nonspecific amplification or low-level splicing artifacts.

**Figure 4:**
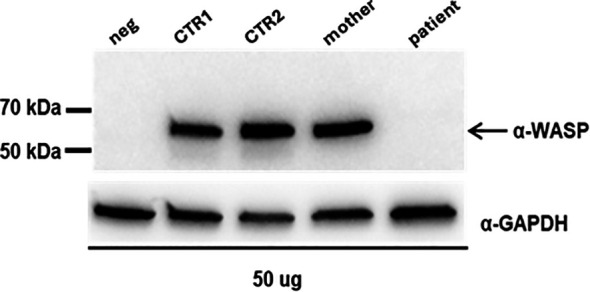
Western blot analysis on proteins extracted from PBMCs; neg: protein lysate from human fibroblasts; CTR1: female, adult; CTR2: male, young. The expression of WASP was quantified by densitometry analysis relative to GAPDH with ImageJ software (CTR1: 1,20; CTR2: 2,05; mother: 1,55).

**Figure 3B** shows the Ct values of EX 8-9 (primers pair Ex8FW-Ex9RV) of the controls and the mother compared to the Ct values of INT 8 of the proband and demonstrates that the transcript levels were similar. The Ct values of INT8 in the proband and EX 8-9 in controls were not normalized against an independent reference gene since the amplification efficiencies of the two primer pairs are approximately equal (data not shown). To further analyze the consequences of the retention of INT8 in the transcript, we performed a Western blot analysis on protein extracted from peripheral PBMCs of the proband, his mother, and the two healthy controls. A single-sharp band of 60 kDa corresponding to the wild-type WASp was detected in the mother and in the two healthy controls, while no band was detected in the proband (**Figure 4**). The Western blot analysis showed that a full-length WASp was not detectable in the affected patient, although the epitope recognized by the anti-WASp antibody used in the analysis is encoded by a region upstream of the site of mutation. As expected, the levels of WASp observed in the carrier mother were similar to those observed in unaffected individuals (CTR1: 1.20; CTR2: 2.05; mother: 1.55).

## Discussion

WAS is a rare genetic disorder that affects the immune system caused by mutations in the *WAS* gene on the X chromosome. More than 300 mutations have been described for *WAS*. Among them, mutations that lead to a complete loss of WASp synthesis are associated with the severe phenotype of WAS; on the other hand, mutations that only partially reduce WASp levels are associated with the milder XLT phenotype (18). This highlights the importance of understanding the molecular basis of mutations in the *WAS* gene in order to predict the clinical outcome and tailor treatment strategies for individual patients. Recent studies have also shown that genetic modifiers and environmental factors can influence the clinical presentation of WAS and XLT, as documented in other IEIs (10, 19, 20). We identified a *WAS* splice site VUS in a 2-month-old boy presenting with thrombocytopenia. Our observation attributes a pathogenic significance to the splice site c.777 + 3_777 + 6delGAGT mutation in *WAS*, revealing a potential role in WAS. Unfortunately, immunological evaluation and clinical features for this patient are only available for the first few months of life, and it was not possible to define the impact of the mutation on the severity of the clinical phenotype since the patient was subsequently lost to follow up. However, based on the total absence of a full-length WASp we could speculate that the patient will develop the severe form of the disease.

Even though it is not always possible to characterize all the variants identified through NGS, the definition of the pathogenic role of the VUS in the NGS era is crucial, especially in cases where there is no clear correlation between the clinical phenotype and the genetic alteration, as in carriers. In fact, the false assignments of VUS pathogenicity can result in incorrect prognostic, therapeutic, and reproductive advice.

## Data Availability

The datasets presented in this study can be found in online repositories. The names of the repository/repositories and accession number(s) can be found below: https://www.ncbi.nlm.nih.gov/genbank/, ERX12289338.

## Supplementary material

Supplementary Table 1 Detailed immune laboratory values of the patient and normal values for age. Reference values adapted from W.T. Shearer, J Allergy Clin Immunol, 2003 (21).
